# Synergistic potential and apoptosis induction of *Bunium persicum* essential oil and its pure components, cuminaldehyde and γ-terpinene, in combination with fluconazole on *Candida albicans* isolates: *in vitro* and *in silico* evaluation

**DOI:** 10.22034/cmm.2025.345248.1636

**Published:** 2025-08-10

**Authors:** Mahsa Asgar, Mehdi Bamorovat, Seyyed Amin Ayatollahi Mosavi, Fatemeh Sharifi, Ehsan Salarkia, Ali Karamoozian, Sanaz Hadizadeh, Setareh Agha Kuchak Afshari

**Affiliations:** 1 Department of Medical Parasitology and Mycology, Afzalipour Faculty of Medicine, Kerman University of Medical Sciences, Kerman, Iran; 2 Leishmaniasis Research Center, Kerman University of Medical Sciences, Kerman, Iran; 3 Research Center of Tropical and Infectious Diseases, Kerman University of Medical Sciences, Kerman, Iran; 4 Department of Biostatistics and Epidemiology, Faculty of Health, Kerman University of Medical Sciences, Kerman, Iran

**Keywords:** *Bunium persicum*, *Candida albicans*, Cuminaldehyde, γ-terpinene, Molecular docking

## Abstract

**Background and Purpose:**

Fluconazole resistance in *Candida* species is on the rise, posing a significant clinical challenge. There is a growing interest in using complementary therapies, especially those from natural sources. This study aimed to evaluate the synergistic and apoptotic effects of *Bunium persicum* essential oil (BPEO) and its two pure components, cuminaldehyde (CA) and γ-terpinene (γ-TPN), combined with fluconazole (FLC) on susceptible and resistant *C. albicans* isolates. Moreover, molecular docking was used to study the interactions between lanosterol 14-alpha-demethylase and each agent.

**Materials and Methods:**

The BPEO was prepared using the Clevenger apparatus and the hydro-distillation method. The *in vitro* antifungal activity was evaluated according to the Clinical and Laboratory Standards Institute guideline (M60). The checkerboard and isobologram assays assessed the interaction between BPEO, CA, γ-TPN, and FLC. The necrotic and apoptotic effects of different agents were analyzed using a flow cytometry assay. An *in-silico* study was performed to examine the receptor-ligand interaction.

**Results:**

The CA showed the lowest minimum inhibitory concentrations and minimum fungicidal concentrations, compared to BPEO and γ-TPN. Statistical analyses indicated significant differences between resistant and sensitive *C. albicans* isolates regarding minimum inhibitory concentration values of BPEO, CA, and γ-TPN. The most synergistic effect was obtained for FLC combined with CA (n=7, 63.6%), followed by BPEO (n=6, 54.5%), and γ-TPN (n=3, 27.2%). Statistical analyses indicated the synergistic effect of FLC in combination with CA was more than γ-TPN (*p*=0.023). Apoptotic indicators confirmed that the tested compounds could cause cell death in yeast cells. Combination of each natural component with FLC resulted in a greater apoptosis effect than each tested agent alone. The docking study indicated that both pure compounds have interactivity with the protein residue of 14α-demethylase.

**Conclusion:**

The results indicated that the synergistic properties of natural products combined with synthetic antifungal agents available in the market could contribute to developing effective therapeutic strategies, particularly in resistant fungal species.

## Introduction

Opportunistic fungal infections, such as candidiasis, have become a growing health problem worldwide, especially in high-risk individuals [ 1 ]. *Candida albicans* is the most important commensal species that can become an opportunistic pathogen and cause many infections [ 2 , 3 ]. Azole derivatives, such as fluconazole (FLC), are among the most common antifungal drugs used to treat *Candida*-related infections. The FLC inhibits the 14α-lanosterol demethylase, the key enzyme in the ergosterol biosynthesis pathway [ 4 ]. Antifungal resistance to the azole derivatives has recently become a serious clinical challenge since the increase in multidrug-resistant patterns could affect mortality rates [ 5 ]. Besides, some currently available antifungal agents have limitations regarding high cost, toxicity, and low drug bioavailability [ 6 , 7 ].  Therefore, exploring and finding novel therapeutic strategies for treating fungal infections that are highly efficacious and have lower side effects is crucial.

Numerous essential oils (EOs) have gained more attention as pivotal medicinal agents [ 8 - 10 ]. Reports have indicated that EOs might be a promising potential drug candidate against various diseases, regarding their pharmacological actions and low toxicity. Among traditional natural products, *Bunium persicum*, which belongs to the Apiaceae family, has received much attention for its beneficial medical effects. It is a significant grassy aromatic plant that naturally grows in some South Asian countries [ 11 ]. Numerous studies have reported that *B. persicum* EO (BPEO) has antioxidant, anti-inflammatory, antimicrobial, antifungal, and antileishmanial properties [ 12 , 13 ]. These promising therapeutic effects of the BPEO have been attributed to the bioactivity of its major components [ 11 ]. Cuminaldehyde (CA; 4-isopropylbenzaldehyde) is one of the major components, which has anticancer, antidiabetic, anti-inflammatory, and antimicrobial activity, and is a food preservative due to its powerful fumigant against phytopathogenic fungi [ 14 - 16 ]. The other substance in the BPEO is gamma-terpinene, which has effective pharmacological properties, such as antimicrobial and antifungal activities [ 17 - 19 ].

In addition, in recent years, combination therapy and synergistic interaction have been well-known as feasible strategies for combating drug resistance [ 20 - 22 ]. The synergy between azoles and EOs could permit the usage of lower doses of these synthetic antifungal agents effectively and safely, presenting a promising pharmaceutical strategy for future management of mycoses. Furthermore, the synergistic properties of combining medicinal plants with available antifungal drugs might enhance antifungal activities. Hence, this study aimed to evaluate the inhibitory activity of the BPEO, compared to its two major pure compounds, including CA and γ-terpinene (γ-TPN), as well as their synergistic effects and apoptosis-inducing potential in combination with FLC against both susceptible and resistant *C. albicans* isolates. Molecular docking was also used to study how Lanosterol 14 alpha-demethylase interacts with two pure components, CA and γ-TPN, in search of antifungal properties.

## Materials and Methods

### Essential oils and compounds

The *B. persicum* seeds were prepared from the market and confirmed by a pharmacognosist in the Department of Pharmacognosy at Kerman University of Medical Sciences, Kerman, Iran (herbarium number KF1141). The BPEO was prepared by the Clevenger apparatus and the hydrodistillation method as described previously, with some modifications [ 13 ]. The obtained BPEO was kept at 20 °C until the experiment. The FLC (Pfizer, Groton, CT, USA) and pure compounds, CA and γ-TPN (Sigma-Aldrich, St. Louis, USA), were bought at 97% purity.

### Fungal isolates

This study was conducted on a standard *C. albicans* strain (ATCC 90028) and 10 clinical *C. albicans* isolates obtained from patients with vulvovaginal candidiasis, which had been previously identified using conventional and molecular methods  [ 23 ].

### In vitro antifungal susceptibility testing

The minimum inhibitory concentration (MIC) of agents was evaluated by the microdilution broth method according to the Clinical and Laboratory Standards Institute (CLSI M60) guideline [ 24 ].  According to the CLSI clinical breakpoint values, *C. albicans* isolates were considered susceptible with MIC ≤ 2 μg/mL and resistant with MIC ≥ 8 μg/mL for FLC [ 24 ]. The FLC and BPEO were prepared at a final concentration of 128-0.125 µg/ml and 500-1.95 µg/ml, respectively. A serial dilution of CA and γ-TPN was prepared from 250-0.24 µg /ml [ 25 , 26 ]. The suspension containing 0.5–2.5 × 10^3^ cells/mL of each *C. albicans* isolate was added to the 96-well microtiter plates containing RPMI-1640 medium (Sigma-Aldrich, USA). The plates were incubated at 35 °C for 24 h and afterward read visually. All experiments were conducted in triplicate using C. parapsilosis ATCC 22019 as the quality control strain. To assess fungicidal activity, 10 μL of cell suspensions from turbidity-free wells were cultured on Sabouraud dextrose agar plates, incubated at 35 ºC for 48 h, and the number of colony-forming units was determined. The minimum fungicidal concentration (MFC) was defined as the lowest concentration at which three or fewer *C. albicans* colonies were observed [ 27 ].

### Assessment of the drug interaction

The microdilution checkerboard assay was performed to assess the fractional inhibitory concentration index (FICI) [ 28 ]. For this purpose, combinatorial effects between FLC and BPEO/CA/γ-TPN were evaluated in the 96-well microtiter plates. Serial two-fold dilutions of each compound, ranging from several dilutions below the MIC to 2 × MIC, were set up. All experiments were tested in duplicate. The FICI was measured as follows: FICI = FIC A + FIC B. The FIC of each agent was determined as follows: FIC A = MIC of drug A in combination / MIC of drug A alone, and FIC B = MIC of drug B in combination / MIC of drug B alone.

The corresponding FIC values of the checkerboard assay were represented graphically by isobologram analysis using Prism software (version 9.0; GraphPad Inc., San Diego, CA). The straight line connecting the intercept points on the x-axis and y-axis signifies an additive interaction between the drugs (FICI=1). Values below this line indicate synergistic (FICI≤0.5) or additive effects (0.5<FICI≤1), while values above suggest indifferent (1<FICI<4) or antagonistic effects (FICI>4) [ 29 ].

### Apoptotic effects analysis

The approach of cell death induced by BPEO, CA, and γ-TPN, alone and in combination with FLC, was investigated using the fluorescein isothiocyanate Annexin V Apoptosis Detection Kit I (BD Biosciences, CA, USA) as previously described [ 30 ]. The *C. albicans* standard strain was exposed to minimum inhibitory concentrations of each tested compound and incubated at 35 °C for 48 h. After incubation, the protoplast of yeast cells was prepared and resuspended in an annexin V binding buffer at a concentration of 1×10^6^ cells/mL. Subsequently, 5 µL of fluorescein isothiocyanate annexin V and Propidium iodide were added to the 100 µL of this solution and kept at room temperature for 15 min. Subsequently, the annexin V binding buffer (400 µL) was used in each tube, and the samples were investigated by a flow cytometer (BD LSRFortessa cell analyzer, Becton Dickinson, USA). Untrained cells were used as controls, and the data obtained were analyzed with FlowJo software (version 10).

### In silico analysis

### Protein-ligand docking

The 3D structure of FLC, CA, γ-TPN, and Lanosterol 14 alpha-demethylase was achieved by the Protein Data Bank (https://www.rcsb.org/) [ 31 ]. The HDOCK server (©Lab of Biophysics and Molecular Modeling, huanglab@hust.edu.cn) utilized peptide-protein docking to determine the receptor association (http://hdock.phys.hust.edu.cn/). The root-mean-square deviation method was applied to assess the difference in the location of the docked peptides. The default parameters were used for all docking runs. The HDOCK server applies an IT Score-PP iterative knowledge-based scoring system (Docking score).

### 2D interaction plots of protein-ligand complexes

The Protein-Ligand Interaction Profiler site (using the Michael Schroeder group at the Biotechnology Center TU Dresden (BIOTEC) at https://academic.oup.com/nar/article/49/W1/W530/6266421) was applied to assess the ligand interaction site (https://plip-tool.biotec.tu-dresden.de/plip-web/plip/index) after the docking procedure [ 32 ]. Afterward, the interactions between the amino acid residues of the target protein and the ligand were determined. Lastly, the 3D shapes of the final interaction were designed using the UCSF Chimera software (version 1.12; University of California, San Francisco, USA).

### Statistical analyses

Statistical analyses were carried out using the generalized estimating equation method based on the Gaussian distribution using logistic regression and linear regression models. An exchangeable correlation structure was used for this method. The SPSS software (version 22.0; IBM, Armonk, NY, USA) was used for data analysis. A *p* value of less than 0.05 was considered statistically significant.

## Results

### In vitro susceptibility testing results

Table 1 shows the MIC and MFC values obtained for BPEO, CA, γ-TPN, and FLC against all *C. albicans* isolates. Based on the antifungal susceptibility testing, clinical *C. albicans* isolates are categorized as different types of FLC-susceptible (n=5) and FLC-resistant (n=5) species. The MIC values for FLC against resistant and susceptible *C. albicans* isolates were within 8-32 and 0.25-1 µg/ml ranges, respectively. The MIC values for BPEO and γ-TPN ranged from 125 to 15.6 µg /ml, while the MIC for CA ranged from 62.5 to 7.8 µg /ml. The MFC values of BPEO and γ-TPN varied from 500 to 62.5 µg /ml, while for CA, it ranged from 250 to 31.25 µg /ml.

**Table 1:** Minimum inhibitory concentration and minimum fungicidal concentration of Bunium persicum essential oil, cuminaldehyde, γ-terpinene, and fluconazole against fluconazole-susceptible and fluconazole-resistant *Candida albicans* isolates.

*Candida albicans* isolates	MIC (µg/ml)				MFC (µg/ml)		
	BPEO	CA	γ-TPN	FLC	BPEO	CA	γ-TPN
R1	125	31.2	125	16	500	125	250
R2	62.5	31.25	62.5	8	250	125	125
R3	62.5	62.5	125	16	250	125	500
R4	125	62.5	125	32	500	250	500
R5	62.5	31.2	31.2	8	125	62.5	125
S1	31.2	31.2	62.5	1	125	62.5	250
S2	31.2	15.6	31.2	0.5	62.5	31.25	62.5
S3	15.6	7.8	31.2	0.5	62.5	31.25	62.5
S4	62.5	31.2	125	1	250	125	500
S5	15.6	15.6	31.2	0.25	62.5	31.25	62.5
ATCC 90028	31.2	7.8	15.6	0.5	62.5	31.25	62.5

MIC: minimum inhibitory concentration; MFC: minimum fungicidal concentration; BPEO: *Bunium persicum* essential oil, CA: cuminaldehyde, γ-TPN: γ-terpinene, FLC: fluconazole, R: resistant, S: susceptible

Table 2 shows the mean MIC values obtained for BPEO, CA, γ-TPN, and FLC. According to the statistical analyses, there is a significant difference between the MIC values of FLZ and those of three natural compounds, including BPEO, CA, and TPN (*p*<0.001). Besides, CA had a lower MIC value than γ-TPN and BPEO. In addition, there were significant differences between resistant and sensitive isolates regarding MIC values of BPEO and active compounds, including CA and γ-TPN. Moreover, the MIC values for FLC-resistant isolates were more than those for sensitive isolates (*p* =0.005).

**Table 2:** Statistical analyses of mean minimum inhibitory concentration and fractional inhibitory concentration index values for *Bunium persicum* essential oil, cuminaldehyde, γ-terpinene, and fluconazole.

	Compounds	MIC		β	% 95 CI for β	*p* value
		Mean ± SD	Median (IQR)			
	**BPEO**	56.80±38.46	62.50 (31.30)	49.19	(32.22, 66.15)	<0.001
	**CA**	29.80±18.76	31.20 (15.65)	22.19	(15.57, 28.81)	<0.001
	**γ-TPN**	69.58±46.03	62.50 (93.80)	61.97	(39.49, 84.44)	<0.001
	**FLC**	7.61±10.14	1.00 (15.50)	0		
		**MIC**				
		Mean ± SD	Median (IQR)	β	% 95 CI for β	*p* value
	**BPEO**	56.80±38.46	62.50 (31.30)	26.99	(10.59, 43.40)	0.001
	**γ-TPN**	69.58±46.03	62.50 (93.80)	39.78	(21.04, 58.51)	<0.001
	**CA**	29.80±18.76	31.20 (15.65)	0		
		MIC				
		**Mean ± SD**	**Median (IQR)**	**β**	**% 95 CI for β**	***p* value**
**Isolates**	**Resistance**	60.24±42.64	62.50 (78.18)	39.08	(11.50, 66.66)	0.005
	**Sensitive**	27.09±29.56	23.40 (28.50)	0		
		**Interpretation**				
		**Synergism**	**Additive**			
		**N (%)**		**OR**	**%95 CI for OR**	***p* value**
**FICI**	**BPEO/FLC**	6 (54.50)	5 (45.50)	0.31	(0.09, 1.02)	0.055
	**CA/FLC**	7 (63.60)	4 (36.40)	0.21	(0.06, 0.81)	0.023
	**γ-TPN/FLC**	3 (27.30)	8 (72.70)	1		

BPEO: *Bunium persicum* essential oil, CA: cuminaldehyde, γ-TPN: γ-terpinene, FLC: fluconazole, β: Beta coefficient, CI: Confidence interval, IQR: interquartile range, FICI: fractional inhibitory concentration index

### Checkerboard assay results

**Figure 1:**
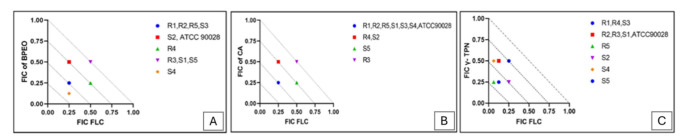
Isobologram analysis of synergistic interaction between fluconazole (FLC) and **A)** *Bunium persicum* essential oil (BPEO), **B)** cuminaldehyde (CA), and **C)** γ-terpinene (γ-TPN) against clinical resistance (R) and susceptible (S) *Candida albicans* isolates. FLC–fractional inhibitory concentration (FIC) values are drafted on the x-axis, while BPEO/CA/γ-TPN–FIC values are drafted on the y-axis.

The checkerboard microtiter test evaluated the most effective combination of components with FLC. The obtained results and interpretation are reported in Table 3. Based on the FICI values, CA was the most effective agent with synergistic activity against seven isolates, while the γ-TPN exhibited the least synergistic activity with only three isolates (n=3). According to the statistical analyses, there was no significant difference between the FICI of FLC/CA and FLC/BPEO regarding synergistic and non-synergistic activity (Table 2). An important difference existed between the FICI of FLC/CA and FLC/γ-TPN. Moreover, the combination of FLC and CA demonstrated a higher odds ratio (OR) for synergistic effect, compared to the combination of FLC and γ-TPN (*p*=0.023). No antagonistic activity was observed, and the isobolographic analysis supported the synergistic interaction between FLC and the tested agents (Figure 1).

**Table 3:** Interactions between Bunium persicum essential oil, cuminaldehyde, γ-terpinene, and fluconazole against *Candida albicans* isolates.

*Candida albicans* isolates	MIC	FICI	Interpretation	MIC	FICI	Interpretation	MIC	FICI	Interpretation
	BPEO/FLC			CA/FLC			γ-TPN/FLC		
R1	31.25/4	0.5	Synergism	7.8/4	0.5	Synergism	31.25/4	0.5	Synergism
R2	15.6/2	0.49992	Synergism	7.8/2	0.5	Synergism	15.6/4	0.74992	Additive
R3	31.25/8	1	Additive	31.25/8	1	Additive	62.5/8	1	Additive
R4	62.5/8	0.75	Additive	31.25/8	0.75	Additive	62.5/8	0.75	Additive
R5	15.6/2	0.49992	Synergism	7.8/2	0.5	Synergism	7.8/2	0.5	Synergism
S1	15.6/0.5	1	Additive	7.8/0.25	0.5	Synergism	31.25/0.5	1	Additive
S2	7.8/0.25	0.75	Additive	7.8/0.125	0.75	Additive	15.6/0.125	0.75	Additive
S3	3.9/0.125	0.5	Synergism	1.95/0.125	0.5	Synergism	7.8/0.125	0.5	Synergism
S4	15.6/0.25	0.4996	Synergism	7.8/0.25	0.5	Synergism	31.25/0.5	0.75	Additive
S5	7.8/0.125	1	Additive	3.9/0.125	0.75	Additive	15.6/0.125	1	Additive
ATCC 90028	7.8/0.125	0.5	Synergism	1.95/0.125	0.5	Synergism	7.8/0.25	1	Additive

MIC: minimum inhibitory concentration, BPEO: *Bunium persicum* essential oil, CA: cuminaldehyde, γ-TPN: γ-terpinene, FLC: fluconazole, FICI: fractional inhibitory concentration index.

### Apoptosis and necrosis result

**Figure 2:**
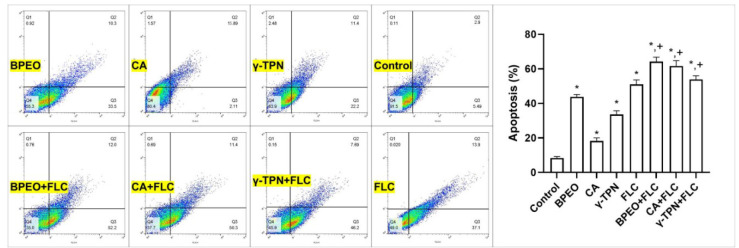
Flow cytometry analysis of *Candida albicans* exposed to the minimum inhibitory concentrations of* Bunium persicum* essential oil (BPEO), cuminaldehyde (CA), γ-terpinene (γ-TPN), and fluconazole (FLC) alone, and the combination of each agent with FLC, compared with untreated control after 48 h incubation. Q1: necrosis (annexin V-/propidium iodide+), Q2: late apoptosis (annexin V+/ propidium iodide+), Q3: early apoptosis (annexin V+/PI-), and Q4: viable cells (annexin V-/ propidium iodide-). Bars show the mean ± standard deviation of viability rates (n=3). Significant differences compared to the control group are marked with an asterisk (*), while differences between combination and single usage of each agent are indicated with a plus sign (+) (*p<0.001).

The flow cytometry results showed the apoptosis and necrosis effects on the *C. albicans* isolate exposed to each agent alone and in combination with FLC (Figure 2). The sum of early and late apoptosis percentages was measured for each sample to calculate the level of apoptotic cells. Briefly, when BPEO, CA, and γ-TPN were used alone, they induced apoptosis (primary/final) in 43.8%, 17.91%, and 33.6% of the yeast cells, respectively. Furthermore, the flow cytometry results revealed that the combination of all natural components with FLC had a significant apoptotic effect on the *C. albicans* isolate, compared to using each agent alone (*p*<0.001).

### In silico docking results

**Figure 3:**
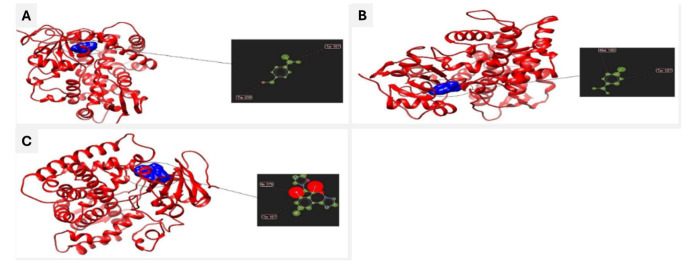
The 3D interactions of Lanosterol 14 alpha-demethylase and **A)** cuminaldehyde, **B)** γ-terpinene, and **C)** fluconazole using Chimera software (target cavities show the interaction of the ligand with residues where the amino acids are involved).

**Figure 4:**
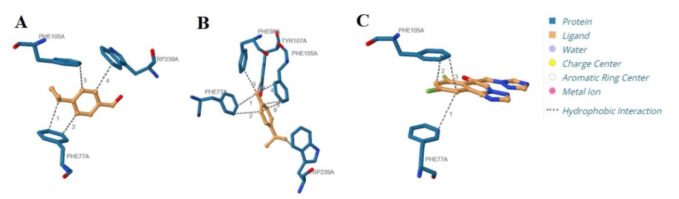
Ligands of **A)** cuminaldehyde/Lanosterol 14 alpha-demethylase, **B)** γ-terpinene/Lanosterol 14 alpha-demethylase, and **C)** fluconazole/Lanosterol 14 alpha-demethylase by the Protein-Ligand Interaction Profiler server.

Figure 3 illustrates the 3D interactions of CA, γ-TPN, and FLC with Lanosterol 14 alpha-demethylase, using Chimera software. As shown in Figure 3, CA can bind to Lanosterol 14 alpha-demethylase at active site residues Tyrosine107 and Tryptophan 239 (Figure 3A), γ-TPN at active site residues Tyrosine107 and Methionine100 (Figure 3B), and FLC at active site residues Tyrosine107 and Isoleucine379 (Figure 3C). In addition, the docking scores of CA, γ-TPN, and FLC with lanosterol 14 alpha-demethylase were -82.40, -77.19, and -156.82, respectively. Furthermore, the results of Ligand root-mean-square deviation (Å) were 80.59, 80.00, and 75.57, respectively. The amino acids involved in the interaction of CA, γ-TPN, and FLC core pocket with Lanosterol 14 alpha-demethylase are shown in Figure 4. It must be mentioned that Tyr107 is the common amino acid affirmed with Molegro Molecular Viewer 2.5.0 (Molegro ApS, Aarhus, Denmark). Table 4 displays ligands of hydrogen bonds and hydrophobic interactions.

**Table 4:** 2D interaction plot analysis of hydrophobic interactions of cuminaldehyde, gamma-terpinene, and fluconazole with lanosterol 14 alpha-demethylase.

	Residue	Aminoacids	Distance	Ligand Atom	Protein Atom
Cuminaldehyde	77A	PHE	3.41	3592	157
	77A	PHE	3.34	3596	156
	105A	PHE	3.45	3597	382
	239A	TRP	3.35	3599	1438
Gamma Terpinene	77A	PHE	3.54	3600	157
	77A	PHE	3.98	3594	156
	98A	PHE	3.44	3600	329
	105A	PHE	3.54	3600	37
	105A	PHE	3.62	3596	381
	105A	PHE	3.36	3594	382
	107A	TYR	3.37	3600	398
	239A	TRP	3.20	3598	1436
Fluconazole	77A	PHE	3.99	3607	156
	105A	PHE	3.86	3606	381
	105A	PHE	3.78	3607	382

## Discussion

Treatment of *Candida* infections due to the drug-resistant species is becoming a crucial concern worldwide [ 33 ]. Besides, since higher treatment doses of currently available antifungal agents can lead to various adverse effects, choosing the proper alternative therapies is crucial. Over recent years, traditional herbal medicine has been widely useful in fungal infection therapy due to its broad-spectrum activity and low toxicity [ 10 ]. *Bunium persicum* EO and its bioactive components have been used due to their promising medical activities all around the world [ 13 , 34 ]. In this regard, the present research examined the *in vitro* antifungal activity of BPEO, compared to its main components, CA and γ-TPN, against FLC-susceptible and -resistant *C. albicans* isolates.

The results confirmed the inhibitory effect of BPEO and its two pure components against both susceptible and resistant *C. albicans* isolates. According to the statistical analyses, CA showed the lowest MICs and MFCs compared to BPEO and γ-TPN in the present study. Furthermore, numerous previous studies have reported antimicrobial and antifungal activities of CA [ 35 , 36 ]. Sekine *et al*. evaluated the antifungal effects of different volatile compounds against phytopathogenic fungi and reported that *B. persicum* had the most antifungal activity [ 12 ]. In addition, their findings displayed CA as the main antifungal compound with stronger antifungal activity against *F. oxysporum*, compared to other compounds in black Zira [ 12 ]. Findings of the present study demonstrated the highest MICs for γ-TPN, which is consistent with those of a study performed by Mandras *et al*. They reported no anticandidal effect of γ-TPN among the tested EOs and their bioactive pure compounds, while α-pinene displayed effective anticandidal activity against non-albicans *Candida* isolates [ 37 ]. Another study investigated the potential of 50 EOs against *Candida* biofilms. In this work, γ-TPN exhibited a moderate or weak correlation to biofilm inhibition [ 38 ]. According to the findings, it is inferred that the antifungal activity of BPEO in the present study was mainly due to the presence of CA.

To the best of our knowledge, no studies have been performed on the synergistic and apoptotic effects of BPEO and its pure bioactive compounds in combination with FLC against clinically susceptible and resistant *C. albicans* isolates. In the present study, the FICI results indicated that CA combined with FLC had a synergistic effect against seven *C. albicans* strains, with no significant differences between the susceptible and resistant strains. The synergistic effect was obtained in three isolates when γ-TPN was combined with FLC. Moreover, CA showed inhibitory effects in combination with FLC at concentrations lower than their individual MIC values. Some previous studies have confirmed the synergistic activity of EOs and their main bioactive compounds in combination [ 36 , 39 ]. Touil *et al*. reported that combining CA and carvacrol caused synergistic interactions in the most tested *C. albicans* strains [ 36 ].

In the current study, flow cytometry results showed that BPEO, CA, and γ-TPN had apoptosis and necrosis effects. More remarkably, the combination of these natural compounds with FLC showed higher apoptotic effects than each tested agent alone. Some studies have reported the necrotic and apoptotic effects of other natural antimicrobial compounds on *Candida* species [ 40 ]. Results of the present study are consistent with those of another study, which reported a significantly greater apoptosis rate in the combination group, compared to the other groups [ 40 ]. Generally, the processes of apoptosis and necrosis can be induced by different pathways. Since there is no data about the probable induction mechanisms of apoptosis and necrosis by *B. persicum* and its derivatives, future research is necessary to consider these compounds as suitable new antimycotic agents.

In addition, a computational study was conducted in this research to determine the mechanism of synergistic interaction. As mentioned in the results section, natural composition exhibits a synergistic effect via multiple targets through binding to Lanosterol 14 alpha-demethylase with various active site residues. Lanosterol 14α-demethylase is a crucial enzyme in the ergosterol biosynthesis pathway, which is vital for maintaining the integrity of fungal cell membranes. Inhibition of this enzyme disrupts the production of ergosterol, which is the primary mechanism by which azole antifungals, such as FLC, operate. Fluconazole is commonly used to treat *Candida* infections. However, with the rising prevalence of FLC-resistant *Candida* strains, there is an urgent need to explore alternative or adjunctive compounds that can either inhibit this enzyme or enhance the effectiveness of the existing azole antifungals. Molecular modeling studies suggest that most natural compounds demonstrate antifungal activity by inhibiting the 14α-demethylase enzyme [ 41 ]. The findings indicate that the presence of a hydroxyl group significantly enhances the activity of these compounds.

The highlighted amino acids in Lanosterol 14α-demethylase—specifically Tyr107, Trp239, Met100, and Ile379—were identified as crucial based on molecular docking analysis results. These residues were selected since they formed direct interactions with the docked ligands (CA, γ-TPN, and FLC), as determined by the Protein-Ligand Interaction Profiler tool following the docking procedure. Their functional relevance is supported by their location within or near the active site of the enzyme, which is vital for substrate binding and catalysis. For example, Tyr107 and Trp239 often help stabilize ligand binding through hydrogen bonds or hydrophobic interactions, while residues, like Met100 and Ile379, contribute to the structural configuration of the active site, impacting the function of the enzyme and how it interacts with inhibitors [ 42 , 43 ].

The docking analysis performed in the present study revealed that CA and γ-TPN interact with Lanosterol 14α-demethylase at sites distinct from the FLC binding pocket, suggesting a potential allosteric or structural modulatory mechanism. Such interactions may induce conformational changes or destabilize the enzyme, thereby impairing its activity and enhancing the efficacy of FLC. This mechanistic hypothesis is consistent with the in vitro findings of synergistic effects in the present research and warrants further investigation through mutagenesis or biophysical studies. Nevertheless, since the in vivo interaction mode of the two molecules is affected by various physiological factors, further studies are essential to confirm the present findings.

## Conclusion

In conclusion, the findings displayed that CA has more effective activity than the EO of *B. persicum* and γ-TPN against both FLC-resistant and -susceptible *C. albicans* isolates. Furthermore, the synergistic effects due to the combination of these natural products with available synthetic antifungal agents could contribute to the optimal inhibition strategies, particularly in resistant fungal strains. However, the possible mechanism of action of *B. persicum* EO and its pure compounds alone and combined with commercial antifungal drugs requires further studies to confirm their potential for clinical application.
